# Birthing Between the “Traditional” and the “Modern”: *DāĪ* Practices and Childbearing Women’s Choices During COVID-19 in Pakistan

**DOI:** 10.3389/fsoc.2021.622223

**Published:** 2021-05-07

**Authors:** Inayat Ali, Salma Sadique, Shahbaz Ali, Robbie Davis-Floyd

**Affiliations:** ^1^ Department of Social and Cultural Anthropology, University of Vienna, Vienna, VIE, Austria; ^2^ Department of Community Health Sciences, Peoples University of Medical and Health Sciences for Women (PUMHSW), Sindh, Pakistan; ^3^ Independent Researcher, Islamabad, Pakistan; ^4^ Department of Anthropology, Rice University, Houston, TX, United States

**Keywords:** COVID-19, traditional midwives, Dāī, maternity care, Sindh Province, Pakistan

## Abstract

Pregnancy and birth are biological phenomena that carry heavy cultural overlays, and pregnant and birthing women need care and attention during both ordinary and extraordinary times. Most Pakistani pregnant women now go to doctors and hospitals for their perinatal care. Yet traditional community midwives, called *DāĪ* in the singular and *Dāyūn* in the plural, still attend 24% of all Pakistani births, primarily in rural areas. In this article, *via* data collected from 16 interviews—5 with Dāyūn and 11 with mothers, we explore a maternity care system in tension between the past and the present, the DāĪ and the doctor. We ask, what does the maternity care provided by the Dāyūn look like during times of normalcy, and how does it differ during COVID-19? We look at the roles the DāĪ has traditionally performed and how these roles have been changing, both in ordinary and in Covidian circumstances. Presenting the words of the Dāyūn we interviewed, all from Pakistan’s Sindh Province, we demonstrate their practices and show that these have not changed during this present pandemic, as these Dāyūn*,* like many others in Sindh Province, do not believe that COVID-19 is real—or are at least suspect that it is not. To contextualize the Dāyūn, we also briefly present local mother’s perceptions of the Dāyūn in their regions, which vary between extremely positive and extremely negative. Employing the theoretical frameworks of “authoritative knowledge” and of critical medical anthropology, we highlight the dominance of “modern” biomedicine over “traditional” healthcare systems and its effects on the Dāyūn and their roles within their communities. Positioning this article within Pakistan’s national profile, we propose formally training and institutionalizing the Dāyūn in order to alleviate the overwhelming burdens that pandemics—present and future—place on this country’s fragile maternity care system, to give mothers more—and more viable—options at *all* times, and to counterbalance the rising tide of biomedical hegemony over pregnancy and birth.

## Introduction

Pregnancy is a biological phenomenon that is always, as Brigitte Jordan (1993) famously noted, “culturally marked and shaped.” Thus many social scientists, and especially anthropologists, have paid significant attention to this cultural marking and shaping of birth (see for examples Davis-Floyd and Sargent, 1997; Ram and Jolly, 1998; De Vries et al., 2001; Lukere and Jolly, 2002; Davis-Floyd 2018; Cheyney and Davis-Floyd, 2019; Ali et al., 2020). How this biological phenomenon is affected during the challenges of COVID-19 (C-19) is currently being widely researched, as indicated in the articles in this Special Issue and many others. Yet little of that research to date has focused on the challenges C-19 poses to traditional midwives, and particularly in Pakistan, where they are known as *Dāyūn* in the plural and *Dāī* in the singular.

Empirically situated within Pakistan, mostly Sindh Province, this article aims to present: 1) the perceptions and practices of Dāyūn during ordinary times; 2) the Dāyūns’ perceptions of and (non)practices around COVID-19; and 3) local mothers’ perceptions of the Dāyūn working in these mothers’ regions.

## Methods and Materials

### Research Design

The article builds on various data resources, mainly ethnographic observations and fieldwork conducted during the COVID-19 pandemic, which was initially reported in Pakistan in February 2020. From March to October 2020, we adopted a qualitative research study design with an interview guide to gather data, study and comprehend the perspectives of Dāyūn and mothers dealing with the pandemic. Reproductive anthropologist Robbie Davis-Floyd added her considerable international expertise in midwifery and birth to generating the questions we asked the Dāyūn and worked with Inayat Ali to prepare the interview guide for the questions asked of the Dāyūn and the mothers.

### Participants and Sampling

We used the purposive sampling method to select interlocutors. We conducted 16 virtual and in-person interviews: 5 with Dāyūn (from Sindh Province) and 11 with mothers who have used these midwives’ services. All interlocutors were informed about the project and asked to give their consent. Since the first three authors conducted their previous ethnographic research in Sindh Province, they were aware of the Dāyūn but did not know them personally. We reached them via our pre-existing social contacts—family and acquaintances. The data generated from these five interviews proved sufficient for this article, as we reached saturation in terms of themes and information provided. These five Dāyūn are highly representative of the other Dāyūn practicing in Sindh Province because they all practice according to the same cultural traditions and share in the same belief system/worldview. It is impossible for us to guess their number, given that there is no official record available.

### Data Collection

Using the interview guide, we focused our interviews with the Dāyūn on our central questions for them: How do they practice in ordinary times, and how have they dealt with the effects of the COVID-19 pandemic in Pakistan? Each specific question we asked is listed below above their responses to that question. Additionally, we carried out content and document analysis of news reports and various surveys, mainly government reports, to contextualize the pandemic in Pakistan as background for understanding our interview results. Using the Sindhi language, the first three authors conducted the interviews with the Dāyūn and the mothers in person at homes and as telephone conversations. We conducted the interviews with the mothers in the same ways. Our primary questions for them had to do with their perceptions of the Dāyūn and their practices. Later, we first three authors transcribed the data verbatim into English.

This article forms part of a larger project on COVID-19 in Pakistan, principally led by Inayat Ali and approved by the National Bioethics Committee of Pakistan (reference No. 4-87/NBC-471-COVID-19-09/20/). The names of interlocutors have been anonymized to maintain confidentiality. Moreover, the three authors also draw on their previous long-term ethnographic fieldwork in Pakistan, mainly in Sindh Province—Inayat Ali (2005-present), Salma Sadique (2013-present), and Shahbaz Ali (2012-present)—to supply qualitative data as background information.

### Data Analysis

Data analysis was ongoing from the first interview. Data gathered from interviews and media were subjected to content analysis. Authors Inayat Ali, Salma Sadique and Shahbaz Ali continually read and re-read the obtained data to gain familiarity with it and allow for iteration. During these processes, salient themes were identified. The first three authors worked on the first draft. Then Inayat Ali and Davis-Floyd revised the article to refine the highlighted themes and played central roles in this article’s crafting, most especially the discussion and analysis sections. The data obtained were eventually organized in terms of questions and interlocutors’ verbatim responses, which we present below.

## Theoretical Frameworks: Authoritative Knowledge and Critical Medical Anthropology

Health and ill-health are embedded in socio-cultural, political, economic and ideological structures and processes, as these factors influence specific health-seeking attitudes and behaviors. To understand a health-seeking action, it is essential to situate it within these contexts. Paying close attention to such health-seeking attitudes and behaviors shows subtle and complicated power dynamics at play not only in individuals but also in systems of knowledges and practices. Some knowledge systems come to dominate all others (Jordan, 1993). That is the case with the Western biomedical system, which has managed to achieve near-global hegemony and against which all other healthcare knowledge systems are evaluated. Thus such knowledge systems are often called “complementary,” or “alternative”—meaning complementary or alternative to biomedicine, despite the fact that these systems may be primary sources of authoritative knowledge in their societies.

A bit of history is relevant here. When the British colonizers invaded old India (which then included what is now Pakistan), they affected every institution, including the extant medical systems, by enforcing their ideas, styles and methods of healthcare and what is now termed “biomedicine.” Biomedicine (also referred to as “Western medicine,” “conventional medicine,” or “mainstream medicine”) focuses on human biology and physiology in research and clinical practice, with treatment administered via formally trained doctors, nurses, and other such licensed practitioners (Banerji, 1974; Zaidi, 1988; Gaines and Davis-Floyd, 2004). As a result, the pre-existing local medical systems were governmentally neglected, rendered unable to retain their previous cultural authority, and re-considered as “alternative” (Ali, 2020b).

In lower resource countries, such alternative knowledge systems are usually called “traditional,” and contrasted with “modern,” meaning “biomedical,” systems. Therefore, we employ Brigitte Jordan’s (1993, 1997) helpful and widely used concept of *authoritative knowledge* as one of our analytical entry points. By “authoritative knowledge,” Jordan specifically did not mean only the knowledge of “the authorities,” though her concept is often used in this way. She meant any knowledge system considered authoritative by its users, on the basis of which people make decisions and take actions. Hence, authoritative knowledge can be held by individuals or entire communities; it can be the knowledge of the authorities or can be communally shared within cultures or groups.

Our ethnographic research for the prior projects described above shows that in Pakistan’s Sindh Province, many rural people reject the notion that the knowledge held by governmental or by biomedical practitioners is always authoritative, especially during COVID-19. They prefer instead to rely on shared community ways of knowing, which in their case tend to insist that COVID-19 is not a real disease but rather a government plot to gain more foreign aid. For context, we note that this suspicion and/or outright rejection of governmental and “Western” authoritative knowledge have also specifically emerged in relation to vaccines and vaccination campaigns (Ali, 2020b; Ali, 2020c). People in Pakistan, including those of Sindh Province, have demonstrated extreme resentment and rejection of both—ever since the fake vaccination campaign carried out in 2011 by the US CIA to discover the whereabouts of Osama bin Ladin. These rural people also are suspicious of the ingredients of vaccines, given that some (malnourished and stunted) children have become extremely ill or have died from vaccine administration. Thus, since 2011, many vaccinators and their security guards have been attacked, and over 100 vaccinators have been killed (ibid).

We provide this information to illustrate some of the many reasons why the rural peoples of Sindh Province are so suspicious of governmental authoritative knowledge about COVID-19 and believe that it is a government “plot.” The Dāyūn interviewed for this article share these same suspicions, which for them sometimes do and sometimes do not extend to the biomedical care of pregnant and birthing women. These midwives often prefer to trust their own authoritative knowledge about birth, including during COVID-19. Yet they are willing to transfer their clients to biomedical facilities when they face complications they cannot handle, considering the practitioners in these facilities to be the ultimate sources of authoritative knowledge (AK) about birth—though not for normal, uncomplicated births or for birth complications such as breech presentations, which they believe they are competent to handle themselves.

In Pakistan, there are significant differences between the multiple medical pluralisms that are considered meaningful sources of AK among the populace and the national healthcare system, which the government and biomedical practitioners have successfully made into the primary source of AK about injuries and diseases (Ali, 2020b). These other highly culturally regarded sources of medical (not including “biomedical”) authoritative knowledge include Ayurveda, which some believe had its earliest origins in the region (3300–1300 BCE), while others place the origin of the first Ayurvedic text at around 550 CE), *Unami-Tib* (see below) and various folk medical knowledge systems, which are considered by the government not only as “alternative” but also as inferior (ibid). This national-level authority provided to Western-style biomedicine results in various structured forms of disparities, in which certain countries of the Global North and their cultures are globally dominant, and thus their “modern” standards of living and their systems of AK—most especially biomedical systems—are supposed to be striven for by low-resource, marginalized, and “pre-modern” countries.

This striving is expected by international development agencies such as the World Health Organization (WHO), the United Nations International Children's Emergency Fund (UNICEF), and the United States Agency for International Development (USAID), which tend to make the colonialist assumption that the more modern and biomedical they can help a low-resource country to become, the better off it will be. Thus the governments of low-income countries like Pakistan are urged to extensively support biomedicalization, and are given no support for their own “traditional” healthcare systems such as, again, Ayurveda and Unanī-Tib, which is an ancient system of medicine based on the teachings of Hippocrates and Galen, subsequently developed into a comprehensive and integrative healthcare system by Arabic and other practitioners. It is now, like the more well-known Ayurvedic system, also practiced extensively in Western nations and considered to be a form of holistic, “integrative” (a term used by holistic practitioners to contradict and critique the use of the terms “complementary” and “alternative”) healing based on supporting and augmenting the body’s own ability to heal itself. Although Unanī-Tib is now slightly institutionalized in Pakistan, and is taught in several university programs in India, it confronts significant issues in terms of resources to conduct research and operationalize it as a valued source of AK. For one example of such issues, most biomedical practitioners consider both Ayurveda and Unanī-Tib to be “quackery,” as they also do for homeopathy and various other non-biomedical healing modalities (Davis-Floyd and St John 1998; Ali, 2020b). Another significant issue in Pakistan is who chooses to be a biomedical doctor (*Ddākdār*): usually, due to the high status, prestige, and financial benefits attached to the biomedical profession, many who wish to practice some form of medicine do not opt for Unanī-Tib or for Ayurveda, but for Western biomedicine and its system of authoritative knowledge.

The Dāyūn tend to follow the principles of Indigenous or “traditional” healthcare systems and mostly recommend medicines from Ayurveda, Unanī-Tib, or made by themselves at home. They used to be prestigious members of their communities, and some still are, as we discuss below. However, their status has significantly decreased in the country overall due to the high value now placed on biomedical maternity care. A few decades ago, the government of Pakistan, following guidelines from WHO, started a “lady health worker” (LHW) program to biomedically train local women to attend births. Because of this training, an LHW has more prestige and power than a Dāī, especially in those social circles that are economically well-off and have formal education.

As we shall show, a Dāī is still honorifically termed and perceived as a “mother” in villages, where biomedical healthcare facilities are still either unaffordable, inaccessible, or ineffective despite substantial attention from the government and global stakeholders. In such settings, a Dāī plays a pivotal role that can be seen against the backdrop of the structured socio-cultural, economic, and (geo-)political disparities prevailing in Pakistan. How such disparities shape health-seeking behaviors has been extensively studied and theorized by critical medical anthropologists (see Farmer, 1996; Briggs and Nichter, 2009; Biehl, 2016; Singer and Baer, 2018; Ali and Ali, 2020; Ali, 2020b) while other anthropologists have used authoritative knowledge as an effective analytical entry point and theoretical framework (for multiple examples, see Davis-Floyd and Sargent, 1997).

Herein we utilize both of these—critical medical anthropology (CMA) and the concept of authoritative knowledge (AK)—to form a cohesive theoretical framework for our data presentation and analysis and in order to illustrate the authoritative hegemony of Pakistani biomedicine, the institutionalized forms of disparities that affect the practices of the Dāyūn, and the conflicts and similarities between their systems of AK and that of Pakistani biomedicine. To accomplish the latter, below we examine the practices of the Dāyūn in biomedical terms and in terms of scientific evidence. (These two are discrepant; see Davis-Floyd, 2003a; Miller et al., 2016 for thorough analyses of the lack of a scientific evidence base for standard obstetric procedures for labor and birth).

## Pakistan’s Profile: Demographic, Sociocultural and Health-Seeking Behaviors

The fifth most populated country in the world, with a total population of 22.23 million, Pakistan reports a total fertility rate of 3.6 births per woman (National Institute of Population Studies (NIPS) [Pakistan] and ICF, 2019)—higher than those of its neighboring countries. On average, mothers living in rural areas bear one child more than mothers in urban areas (3.9 vs. 2.9 births per woman) (ibid.) To provide maternal and child health care, Pakistan has Emergency Obstetric and Neonatal Care (EmONC) services in 275 hospitals and 550 health facilities, and family planning services in every health facility. Despite all that, the maternal mortality rate (MMR) is 170/100,000, still remarkably high compared to the other countries in the region (Government of Pakistan, 2019). Skilled practitioners (doctors, nurses, midwives, and female health visitors) provide antenatal care (ANC). Skilled attendant deliveries, the vast majority of which are assisted by doctors, have increased from 26% in 1990–91 to 69% in 2017–18, while the proportion of births attended by Dāyūn has concomitantly decreased from 41% in 1990–91 to 24% in 2017–18 (NIPS and ICF, 2019). According to one study published in 2008, around 70% of births took place at home at that time, usually assisted by a Dāī (Bhutta et al., 2008). Although there are no specific current statistics on the number of births attended by a Dāī in Sindh, studies report that it is substantial (Mcnojia et al., 2020).

The significant overall decrease in utilization of the Dāyūn’s services can best be seen as a result of the Pakistani government’s desire to phase out their traditional or Indigenous midwives—referred to in the international agency lexicon as “traditional birth attendants”—by providing more hospitals and smaller maternity homes/clinics, as is also being done in many other low-income countries. Anthropologists in general strongly prefer the term “traditional midwife” to acknowledge these practitioners’ recognition in their communities as midwives (Davis-Floyd, 2018). In Pakistan, they practice almost exclusively in rural areas. Almost 4 decades ago, as in other countries, Dāī training programs were started in Pakistan, but were ultimately discontinued (Bhutta et al., 2008) because these trainings did not result in lowered maternal mortality rates. Yet these trainings were not offered in culturally appropriate ways, but rather in didactic, biomedical ways that failed to take into account and work with the culturally embedded authoritative knowledge of the Dāyūn. Additionally, these programs were not based on experiential learning—the primary learning mode of the Dāyūn, but rather on didactic, biomedical ways of teaching to which the Dāyūn could not relate (see Jordan, 1993; Cheyney et al., 2021 on the importance of experiential learning, which is the primary learning mode of all humans, including obstetricians and other medical personnel).

### The Healthcare System in Pakistan

In Pakistan, medical pluralism prevails; as partially noted above, it includes biomedicine, Ayurveda, Unanī-Tib, homemade remedies and verbal healing *via* prayers and supplications (Ali, 2020b). Despite substantial efforts by the Pakistani government to shape people’s perceptions and practices related to perinatal care, there is still a dearth of required facilities and skilled providers (Ali and Ali, 2020). Its biomedical system contains dispensaries, basic health units (BHUs), rural health centers (RHCs), and referral hospitals called District Headquarters (DHQs) and Tehsil Headquarters (THQs). Pakistan has approximately 1,300 public sector hospitals, 5,530 BHUs, 700 RHCs, and 5,680 dispensaries, (which are the smallest primary biomedical healthcare units in Pakistan) (ibid.). There is one doctor per around 970 people, one dentist per around 9,450, and one hospital bed per 1,610 people (ibid)—resulting in extreme hospital and practitioner overwhelm when COVID-19 patients came flooding in, especially in urban areas. There is a significant difference in healthcare provision between rural and urban areas; urban areas have enough and more proper facilities than rural populations—a phenomenon labeled “urban bias” in healthcare policies (Zaidi, 1985; Ali, 2020b). Moreover, biomedical facilities in rural areas have inadequate and less qualified staff, and THQs and DHQs function poorly, partly due to syndemic corruption (Ali and Ali, 2020; Ali 2020b). And local-level healthcare facilities such as BHUs and dispensaries only function until 14:00 h during the week and are closed on Sundays.

For dealing with reproductive health, as previously noted, the Pakistani government introduced the LHW program in 1994 (Bhutta et al., 2008), into which local women with at least 8 years of formal education were recruited. After receiving 6 months of training to deliver care in the home, each LHW is responsible for about 1,000 people (approximately 200 families). There are over 100,000 LHWs in the country to provide maternal health care, family planning, and primary care in rural areas. Although they do not routinely attend deliveries at home as they have no such training, they maintain birth records, provide promotive and preventive educational services, manage milder illnesses (such as respiratory infections), refer people to healthcare facilities, provide oral polio vaccines, and promote routine immunization (Bhutta et al., 2008). Despite their significant workload, LHWs receive low salaries of approximately US $30 per month (ibid.; Ali 2020b) and often, they do not receive their salaries on time, as the Pakistani media has continually reported over the last decade.

### The Pandemic in Pakistan: A Brief Overview

After reporting its first COVID-19 infection on February 26, 2020, statistics show that the coronavirus had infected over 535,000 Pakistanis and had caused over 11,300 deaths as of January 2021. In Sindh Province, there have been around 241,200 reported cases of COVID-19 and approximately 4,000 deaths. To deal with the outbreak, Pakistan implemented measures such as lockdowns, suspending international and national travel, opening quarantine centers, and deploying armed forces and police to implement these measures (Ali and Ali, 2020). Similar to measles and vaccinations in Pakistan (Ali, 2020a; Ali, 2020b; Ali, 2020c; Ali, 2020d), varying local perceptions of the existence or non-existence of COVID-19 and its causes and treatments resulted in the circulation of various rumors and conspiracy theories that often led to rural people being highly suspicious of, and not following, government-imposed restrictions on travel and gathering in groups, nor preventive measures like mask-wearing and frequent handwashing (Ali, 2020a; Ali, 2020e; Ali and Ali, 2020; Ali et al., 2020).

### Socio-Economic Profiles of the Dāyūn

In this section, before we present the voices of the Dāyūn themselves, we offer their socio-economic profiles, explaining where they live, what type of communities they serve, their roles in those communities (which can be multiple, as they also often serve as healers), and who can become a Dāī. We also describe a few of their practices during pregnancy, as the following section focuses on their roles in birth.

All Dāyūn are female, married or widowed, and with no formal education. To become a Dāī, one must have already given birth to several children and received training from an elder, experienced Dāī—usually a family member, as is true for all of our Dāī interlocutors. In most cases, these learned skills are transferred from their mothers. Their socioeconomic status is low, and their ages usually range from 35 to 80 or even 90 years. Each Dāī has a defined area to practice in, demarcated either by her extended family, ethnic group or subgroup, or geographical access; most geographical areas are inherited. Within their areas, most of the Dāyūn are relatives.

In Sindh Province, the Dāyūn enjoy significant prestige, as indicated by the honorific term by which they are often called—*Dāī Aman* (“Aman” means “mother”). In this patriarchal society where most women cannot leave home alone, a Dāī can easily visit her field without any companion, and she never faces any gender-related harassment. Although this role has decreased over time, the Dāyūn also still often work as healers, especially for *Aurtānnī Bīmārī* (diseases of women). (Due to sociocultural, and primarily religious, reasons, people avoid saying the names of specific diseases pertaining to sexual health, irrespective of gender.)

During pregnancy, the Dāyūn use a specific herbal medicine prepared by the *Hakim* (herbalist), called *Batrīho,* which literally means “32” in the Sindhi language. Although it is available at a *Pansārī* (a grocery store that sells herbal medicines), *Batrīho* is also sold at biomedical pharmacies (locally called “medical stores”). With a mixture of 32 herbs, it is usually used in raw resin or syrup forms for multiple pregnancy issues, including inducing contractions at term, relieving false labor pains, or treating antepartum or postpartum hemorrhage. It is also used in a ground form applied to the vagina with cotton tied with a thread, as the belief is that if a baby is due (full-term), then labor pains will increase; otherwise, false pains will subside (Fatmi et al., 2005). The Dāyūn of Sindh Province attend a substantial number of the births in their allotted regions; the exact number is not known.

## The Dāyūn of Sindh: Views, Practices, the Government, and COVID-19

In what follows, after first introducing them (using anonymized names), we present the voices of the Dāyūn; we will provide discussion and analysis later on. Please note that sometimes, when we are quoting the Dāyūn, at a reviewer’s request Inayat Ali provides the actual Sindhi words used, then offers literal translations and, when needed, their connotational meanings. Here we note that all Dāyūn have worked as a Dāī for many decades, are of low socioeconomic status, and have no formal education. The names we have given them are Sara, Mai Razul, Marvi, Gulan, and Zainab. All are Muslim—the predominant religion in Pakistan—with the exception of Sara, who is Hindu.

Sara is around 65 years of age. She has five children and 16 family members; her family income is around US$50 per month. Mai Razul does not remember her exact age, but guesses that she is around 70. She has nine children and lives in a joint family with 25 members. Their monthly income is around US$60; it increases if she attends some deliveries. For each birth she attends, she earns from US$20 to US$60, depending on the gender of the child, its place in the family order, and the socio-economic condition of the family. If it is a male baby, the first child, and the family is relatively well off, she may receive around US$60, which significantly helps her family with their daily life expenditures. Marvi is 90 years old. She has nine children and lives in a joint family with 18 family members. Her monthly income is around US$60. Gulan is 50 years old. She has three children and lives in a joint family with 15 family members. Her monthly income ranges from US$30 to US$50. Zainab is around 45 years old and lives in a joint family with her six children. She earns around US$40-50 monthly, depending greatly upon the number of deliveries she attends.

### Questions Asked and Answered

#### Where did you receive your midwifery knowledge and training?

Sara received her midwifery education from her aunt, an experienced Daī in that community; Razul from her mother and her husband’s first wife—the three currently work together; Marvi from her grandmother and from her own births: she said, “When I was young, my grandmother often asked me to dissect a hen to see its internal parts (especially the ovary) because this resembles a woman’s internal part^1^. I have also delivered my own babies without the help of anyone.” Gulan learned from her mother, who trained her “to examine the mother during pregnancy, conduct deliveries, and protect the baby.” And Gulan’s mother learned from her mother. Zainab said, “It is our family occupation that we have continued for many generations.”

#### Is the government trying to push you out of practice? Does the government try to make you send women to facilities for birth? Does it help you in any way, such as by offering training?

None of these Dāyūn received any help from the *Hakūmat* (government) in any form, but all said that they wished the government would provide them with additional training. Razul did receive a 5-day training from the NGO Agha Khan, which she said helped her greatly. Zainab pointed out:

Although I do not see any particular movement by the government to push us out directly, some efforts have affected our occupation significantly. In the surroundings, now there are maternity homes where [biomedically] trained women assist in deliveries and earn double the money that we do. Despite massive charges, many economically advantaged women prefer to go there.

And Razul noted, “Nowadays, there is hardly a role of a Dāī in delivery. Since hospitals have been established everywhere, most women prefer hospitals to a Dāī for delivery.” Gulan agreed:

Dāyūn used to play an essential role in the past, but now most of the community members prefer a hospital for delivery. There are a few families who call me to administer a delivery while believing that home is much better than a hospital because giving birth at a hospital is too economically expensive. Many people cannot afford these high hospital expenses because *Ddākdār ta gharīban khy kūhan thā* [Doctors are slaughtering the poor].

#### How many births do you think you have attended in your lifetime? Have any mothers and babies died under your care?

*Sara:* I don’t remember the exact number of births I have attended but there have been many. As I remember, around 15 women and 20 newborns have died at the time of birth. When I observe it is beyond my expertise, I inform the woman’s mother-in-law and husband to take the woman to a hospital for delivery. Sometimes the family does not take her to a hospital because they follow *Pothī* and do not go against it [Pothī is a Hindu religious ritual used to decide whether the delivery should be at a hospital or at home].

*Razul*: I have attended over 2,000 births, and never lost a woman or child during birth.

*Marvi*: I am working as a Dāī for an exceedingly long time. Although I don’t remember the number, I have attended more than a thousand *Wayam* (births). During my 25 years of work, *Char Māyon Āllah Sāin Khy Piyārun ThĪ Wayun* [literally meaning: “four women were loved by Allah.” The connotational meaning is that “four women have died”] at the time of birth, and 10 babies were stillborn (*Katcā bbār’rra*), and five more died after the first week of birth.

*Gulan*: I have attended so many *Wayam* (deliveries), that I don’t remember the exact number. Two mothers died on the second day of delivery and five died after seven days. More than 10 newborns died during birth.

*Zainab*: I have helped in a few thousand deliveries. Around five women have died and some infants.

#### Do you provide prenatal, birth, and postpartum care? What is your care like, what does it consist of? (e.g., massage, palpating the baby to determine size and position, etc?)

*Sara:* Yes, I provide care during *Umaidwarī* [the prenatal period], *Wayam* [birth], and *Ādh-sut* [postpartum care]. I confirm the pregnancy of women. I do prenatal massage and give some herbs for standard vaginal delivery. I primarily provide services during delivery and the first week of a baby’s life.

*Razul:* Some women call us for *Ādh-sut* [postpartum care], for their baby’s body massage, clothes washing, and sometimes for their home chores if they live in a nuclear family. However, if I am called to monitor an *Umaidwārī* [pregnancy], then I give some *Dawā* [medicine] to women such as crystallized sugar and cardamon mixed in milk and black tea, which increase the strength of the labor pains. In the postpartum period, I put *Thāl* [a big steel plate] and three to four bricks on the women’s belly for 1 hour, which helps to bring the *Bbachydānī* [uterus] to its regular place and reduce belly fat.

*Marvi:* I don’t provide such services anymore due to being too old. When I was young, I did *Ādh-sut* [massage] of a woman and child. Presently, I only deliver a baby. Mostly, women come to my home, or I go in the case of an emergency.

*Gulan:* Yes, I provide prenatal, birth, and postpartum care to women. In prenatal care, I provide abdominal massage until delivery. In our community, women in their *Pakan Mahīnan* [third trimester] require oil massage of the entire body. I also provide postpartum care until the *Chathī* [a ritual arranged to name the baby on the sixth day, in which a specific food is cooked, especially sweet rice; relatives participate and give some money^2^]. After delivery I massage the mother’s whole body and wash her *Gandā Kāprā* [literally “dirty clothes” but it signifies clothes with blood that are considered highly impure]. I also massage the newborn during this time. In some cases, I offer this care during *Chālīiho* [40 days] as in our *Sindhī* culture, baby massage is important because it makes the baby’s features beautiful and the baby can sleep easily. On the second day after the birth, I keep an old traditional *Āttā Chākki* [a typical grinder made of coarse stone used to grind wheat flour] on the abdomen of the woman for half an hour, because *Chākki* is heavier and due to this heaviness *Bāchēdāni pēhji jāi tē bēhāndi* [the uterus returns to its usual position], and abdominal fat will not increase. I do this in the morning and ask women not to take breakfast.

*Zainab:* There are a few villages in the surrounding where people call me for assisting in *Wayam*. I pay a visit to these villages often to know about any woman conceiving so that I can guide her right from the beginning. That means I offer prenatal, birth, and postpartum care. I provide massage to the pregnant woman as well as palpating the baby to determine size and position. Many women also go for an ultrasound to determine the gender of the baby.

#### How much do you charge for your care? Can you make a living from your midwifery work?

*Sara:* With no demand for much money, I happily accept whatever is paid. People may pay around [US$5-10] in addition to clothes and sometimes grains or vegetables. It highly depends on how much they have. Sometimes, a mother pays in certain installments. *Bus Hin Kam Sān Asān Jo Guzar Safar Thi Wanjjy Tho*. [This work, although it is not sufficiently paid, helps to make our living].

*Razul:* We do not demand money from them. They give us money of their own will. Some give us [US$10-20] and some pay [US$40]. Yet others may give money, rations as well as clothes to us. The economically well-off women also give us around [US$1-2] daily that helps us in our daily life expenditures. Such an amount is not enough for everything we need, but it helps us lead a satisfying life.

*Marvi:* I am not doing *Dāīpo* [midwifery] for any economic incentives; thus, I don’t ask for money. I just take *Mithāī* [this can be interpreted as sweets or sometimes as money that is paid without demand] on the birth of a baby. I cannot make it as living.

*Gulan:* In my case, it is up to the family how much it pays. Some families just give us new clothes and *Mubārkī* [the amount of money received by relatives at the naming ceremony of an infant] of Chathī and a few women give around [US$20] per delivery, including clothes and rations such as sugar, flour, milk, and rice. We are provided with these goods, are provided with a *Busrī* [a locally prepared sweet bread], and people say: Dāī *Mūnh Mithō Krē* [the Dāī should make her mouth sweet].

*Zainab:* We make this work as our main profession to make our livelihood. Our men do other labor, while we adult women after producing some children work as a Dāī. You know, unmarried girls cannot do this due to our culture. [This can be seen in the entire culture of the country, in which reproduction is a highly private matter, rarely discussed even among one’s close family.] Our older generations train us when we are married and have given birth to a few children.

#### Are you trusted and respected in your community? Or are you regarded as outdated?

*Sara:* Since I am much closer to the community, they have been trusting and respecting me as a *Hakim* [herbalist, local healer] of their community. This is because I have been taking care of them during challenging times without charging them much money. In contrast, [biomedical] *Dākdār* [doctors] charge them significant amounts.

*Marvi:* I am still being trusted and respected in our community. People do love, respect, and take care of me.

*Gulan:* Those who call me, they see me as a trustworthy person and treat me as a mother.

*Zainab*: Yes, we are highly trusted and respected by people. People of all ages and genders call us *Dāī Aman* and they give us prestige equal to their biological mothers. Even those who visit maternity homes to deliver their babies give respect to us, because these older generations were assisted in birth by our older generations.

*Razul:* Being a Dāī, we are still being trusted and respected in our community. They call us *Dāī Aman* [Mother Dāī]. There was a time when Dāī attended all deliveries. Nevertheless, that time is no more, as some families perceive us as *Jāhil* [illiterate people who know nothing].

#### Where do the women you attend give birth? In their own homes with you in attendance or someplace you use for birth? What do you do about cleanliness?

*Sara:* Pregnant mothers come to my hut for delivery, but sometimes I have to go to their home in case of an emergency. When a woman is in labor pain, she sends anyone from the family (especially an older woman, husband, brother, or children) and calls *Jāpo walī* [a term used for the Daī in the Bāggrrī community']. I immediately visit that house for delivery. Houses are not clean like a hospital, but I try to keep that place clean. I wash my hands before and after delivery.

*Razul*: Usually, women give birth in their own homes. They call us before delivery, and then we examine whether we should do the delivery or refer her to a hospital. Before delivery, we make sure that everything should be *Sāf suthrī* [clean]. We also wash our hands before and after delivery.

*Marvi:* Pregnant women visit my home for delivery. In case of an emergency, I go to a woman’s home. I keep the delivery place clean, and I wash my hands with soap twice or thrice before and after delivery.

*Gulan*: When a woman feels some *Sūr* [labor pain], she sends her husband. If the husband is not present during that time, then she sends an elder woman or child to inform me while saying: *Māi or amā khē bār jā sūr Āhin* [the wife or mother is in labor pain]. I immediately visit that house, even overnight or if it is raining. Most deliveries occur in a woman’s house in a separate room, yet sometimes in my hut. Since in our village houses are mostly *Kachā* [made of mud and bricks], it is rather challenging to keep that place clean, but we do our best. I wash my hands twice prior to and post-delivery.

*Zainab*: I regularly visit villages in my surroundings. I know about the *Mahīnā* [months that can be called trimesters] of women and remain attentive about those who are in their *Pakā Mahīnā* [mature or final months]. I am called by the family to their house to administer the delivery.

#### Can you handle birth complications like stuck shoulders and breech birth (baby coming bottom- or feet‐first)?

*Sara*: Yes, I can handle the birth complications, such as *Ūbto Bbār* [breech position of the baby]^3^. In our *Bāggārrī* community, it is obligatory to ask for a goddess through a specific ritual called *Pothī* performed by a *Bhopā* [a religious leader] or an older person at home. Since we practice Hinduism *via* this ritual, we seek supernatural help and permission for delivery: either it will be a standard vaginal delivery or not. If not, then please allow us to visit a doctor. If it is a normal delivery, then we present *Parsād* [a devotional offering made to a god or a goddess that mostly contains food and is shared among people] in the name of our goddess, which may include animals, money, and sweets. Moreover, if the baby comes bottom-first, then I put oil with fingers on the uterus [oil lubrication] and do massage of the abdomen that helps the fetus to change its position immediately.

*Razul*: Before delivery, we examine the pregnant woman to know if there is any *Khatro* [danger or complication] or not. If yes, we recommend our women go to a hospital for delivery. Yet, if during delivery, we have to face such complications, we handle such cases very carefully and deliver the baby.

*Marvi:* If there is *Ūbto Bbār* [breech position], I usually recommend the family bring the mother to a hospital for delivery. Nonetheless, if such cases emerge during delivery, then I deal with these complications carefully.

*Zainab*: Since we are highly trained, based on our experiences and family orientation, I can handle any complications. Nonetheless, if it is truly out of my control, then I accompany the woman to a maternity home to assist the delivery.

*Gulan:* Yes, I know the *Sūbto ya Sidho Bbār* [head-first] and *Ūbto ya Ansidhu Bbār* [breech position of the fetus]. I can handle the birth complication. When there is *Ūbto Bbār* [baby coming bottom-first], I with my crossed fingers, after dipping them in oil, do the abdominal massage of the woman and apply oil lubrication to her uterus. Owing to this, the baby gradually changes its position and standard vaginal delivery happens within half an hour. During such hard situations, I also inform the mothers’ family that the delivery is difficult, and the baby may die in the abdomen. Therefore, there is a need to use my *Dawā* [her prepared medicines] such as white and black glycerin mixed with mustard oil to dip cotton pouches in and to keep them in the woman's uterus. This helps control the adverse effects, especially *Zahar* [poison], caused by a *Mual Bbār* [dead baby] in the *Bbachydānī* of a woman during the delivery.

I also give milk or black tea mixed with castor oil to pregnant women to drink, which increases the labor pain [contraction strength] and helps deliver the baby easily. I know stillbirth *via* abdominal checking. *Pait mē bāār ggōrhō thē wēēndō āhā* [the fetus becomes in a ball or round shape in the uterus]. In that case, I ask the mother about the baby's movement. Moreover, if the delivery complications become worse, then I inform to the mother’s family that I cannot manage it. Despite making many efforts, if I fail to conduct the delivery, then I refer the pregnant woman to a nearby hospital.

In response to Gulan’s comments, we must note that actual hospitals with the required technology, medicine and a trained obstetrician are usually not located in the rural areas of Pakistan, including Sindh Province. The available biomedical facilities are *Wayam Ghar* (maternity homes) run by skilled midwives, whom laypeople call *Dākdār* or *Mandam* (a local version of “madam” used for female birth practitioners) as they cannot differentiate between an obstetrician and a skilled midwife. In some cases, a laboring woman experiencing complications can be brought to a dispensary or a private clinic run by a physician or maybe by a dispenser (a biomedical technician working at a dispensary).

#### What do you do if a baby is born and does not breathe?

All Dāyūn shared the following in common to deal with a baby who does not breathe: to blow *Phuk* (breath) in the baby’s mouth, keep the newborn upside down to let any fluids drain from the mouth, or slap the baby’s back (also to release fluids) until the baby cries. However, a few Dāyūn also described other strategies. Sara added, “I give ash to mix with cow urine in the child’s mouth.” Gulan stated, “We massage the baby with warm mustard oil. If the baby still does not breathe, then we put the *Nārro* [the umbilical cord] after cutting on the *Tawā* [a steel plate used for making chapatti] to heat until it becomes black. Yet, if the measures do not work, then I refer the baby to a nearby hospital to put them on a *Sāh Wārī Machine* [a machine that gives breath, denoting a ventilator].” All these measures help the baby to breathe.

#### How long do you wait to cut the cord? Until it stops pulsing, or right away? What instrument do you use to cut the cord, and do you sterilize it first?

*Sara*: I prefer to cut *Nārro* [the cord] as soon as the baby and placenta are delivered. Most often, I cut the cord with a broken piece of mirror that I think needs not to be sterilized.

*Razul*: We cut *Nārro* between 30 and 60 seconds after birth for improved maternal and infant health and nutrition outcomes. Since I use a new blade or scissor to cut the cord, there is no need to sterilize it.

*Marvi*: Usually, I cut *Nārro* after 1 min while considering that it is better for maternal and infant health. I use a new blade and sometimes an old blade to cut the cord. If I use the old blade, then I put it in boiling water for 5–10 min.

*Gulan*: I immediately cut the cord since a delay in cutting is not good for the mother’s health. I use shaving blades to cut it. I prefer to use a new blade to cut the cord so I don’t need to sterilize it.

*Zainab*: If it is a healthy baby, then I cut the cord right after the birth; otherwise, I can wait for a few minutes to do so [the baby can get more oxygenated blood through the cord]. I always use a new blade.

#### Will you attend the birth of a woman with a previous cesarean?

*Sara*: I have delivered a few women with a previous *operation* [the laypeople in Sindh, including the Dāyūn, use the English word “operation” to mean “cesarean”)^4^.

*Razul*: If a woman with a previous operation faces no complications, we attend the delivery … Once, there was a woman [with a previous cesarean] who was waiting for a vehicle to go to the hospital for delivery but owing to unavailability of transport on time she could not go. At that time, she called me immediately to administer the delivery and by the grace of Almighty Allah, I successfully delivered the baby without any complications.

*Marvi*: I have assisted cases of a woman with a previous operation many times. Yet, I am vigilant to make sure that woman faces no complications; otherwise, I refuse.

*Gulan*: It depends on the baby's position. I have delivered many women successfully who were previously delivered by operation.

*Zainab*: If a woman has an operation history, then I avoid assisting her. Yet I offer my postnatal services, such as massage, washing clothes, and arranging *Chatthī*.

#### Can you deal successfully with postpartum hemorrhage (excessive bleeding after birth)? If yes, how do you handle that? What do you do?

*Sara:* Yes, many times I have dealt with postpartum hemorrhage. I put a cotton pouch in the woman’s uterus to control the bleeding, but if it is excessive and does not stop, then I refer the woman to the hospital for blood transfusion.

*Razul*: To control postpartum hemorrhage, I advise women to walk and to be given *Kuttī* [a local sweet made from dry fruit, honey, butter, crystallized sugar, cardamom, and wheat bread]. If due to physical weakness, the bleeding does not stop, *Kuttī* helps to reduce the excessive blood.

*Marvi:* To control the excessive bleeding, I give the mother cold things such as water or any cold drink. This coldness helps to stop bleeding. If bleeding does not stop, then I refer her to the hospital for treatment.

*Gulan:* If excessive bleeding happens, I massage the upper and lower abdomen of women. After the massage, I put an old but clean rug or cloth in the woman’s uterus to reduce the bleeding.

*Zainab:* I make my best efforts to control the excessive bleeding. But I soon realize if I cannot handle otherwise, I promptly bring the lady to a hospital.

#### Has the number of women coming to you increased since COVID-19? If so, by how much? How many births do you usually attend per month? How many per month since COVID-19?

*Sara*: There is no significant impact of coronavirus to increase the number of women to come for delivery.^5^ It is usual to conduct seven to eight deliveries per month as it was prior to the pandemic.

*Razul*: Most women go to doctors as before. As I am old, I can’t go outside the village for the conduct of delivery. During regular times, I attend four to five deliveries per month. In contrast, during coronavirus, that number has increased to six or seven deliveries since women fear becoming ill due to this virus.

*Marvi*: There is some impact of coronavirus on the number of women to visit me. I usually conduct two to three deliveries per month whilst nowadays it has increased to around six deliveries.

*Gulan*: Due to the coronavirus, many women have preferred home delivery. They share that if they visit a hospital for Wayum [birth], the government may put their name on the list of corona patients and they may die there^6^. Consequently, I have attended over 30 births since the coronavirus started, which is a higher number than usual. Women also think that, if they go to a hospital for delivery, their delivery will only happen by operation instead of a normal delivery.

*Zainab*: It is hard to say. Although the coronavirus has not made a significant difference, I attended to a few more women. In contrast, a few women assisted by me in their previous deliveries and during their pregnancy visited a maternity home to deliver their babies. Those who were my clients and economically poor called me to deliver their babies at their home.

#### Do you feel that COVID-19 is a dangerous disease? Do you ask women to get tested for COVID before birth? What precautions, if any, do you take to keep you and your clients from getting infected? Do you have any access to personal protective equipment (PPE)? Does the government help you with that at all, or support you in any way?

*Sara*: I heard for many months that coronavirus is a dangerous disease, and those infected can die. Honestly, no one has been infected with this virus in our community. Therefore, it is not a disease, but these are only *Afwāhūn* (rumors) by the government to get funds from other countries. I don’t follow the government recommended measures. Nevertheless, I wash my hands prior to and after delivery. There is neither availability of *Hifāzatī Sāmān* ^7^ nor provision by the government. We even don’t receive any funds during normal times under Ihsās and the Benazir Income Support Program [BISP^8^] by the government. The government only gives funds to Muslim communities [and we are Hindu].

*Razul*: Coronavirus is just an *Afwāh* [rumor]. That is why we do not wear a mask during the delivery, but yes, we wash our hands, which we also do during normal times. We do not have any kind of PPE. The available rags and equipment at home are used during the delivery.

*Marvi*: I am skeptical of whether coronavirus exists or not. Without following the specific measures recommended by the government, I thoroughly wash my hands. Also, the government has not provided me with any Hifāzatī Sāmān [PPE].

*Gulan*: Although I have heard that coronavirus is a dangerous disease, I have not seen any infected person in my village. I believe that it is propaganda by non-Muslim people. I don’t wear any mask. Actually, no one in our village wears a mask or keeps a physical distance. Everyone in our village has participated in all gatherings such as religious processions, marriage, and funerals. We don’t have any fear of corona. We live our lives as usual. Yet, I am delighted that due to fear of coronavirus, many women have visited me for delivery. To attend them, I have adopted the usual measures—cleaning the place and washing my hands. It would make no sense to ask a woman to wear a mask when she is going through enormous pain and she needs to be able to breathe. I have not received any support from the government, such as Hifāzatī Sāmān [PPE kits]. To practice safe delivery at home, I buy blades, scissors and threads myself from the bazaar.

*Zainab*: There are many stories circulating about this *Wabā* [infectious disease] of coronavirus. Some say it is true and some refute it. I maintain the same preventive measures that I have been used to—washing my hands, cleaning the space where the woman will deliver the baby, giving a warm bath to the baby. Normally, when a woman is in labor pain, she stays in a room along with a few women—mostly her mother or mother-in-law, or grandmother—and her news of delivery is kept secret. We believe that making it public makes the birth complicated. Often, when I was assisting a delivery, we were a few women there and we kept our hands washed. No, I neither received any Hifāzatī Sāmān (PPE) nor I could afford to buy it.

#### Has the government at all recognized the value of home births during the coronavirus, or do they still want all women to go to clinics or hospitals for birth?

*Razul*: The government has no role in deciding or implementing a decision to deliver at home or hospital. The pregnant woman or her family usually decides whether she should go to a hospital or call a Dāī for the delivery. No woman who delivered a baby during COVID-19 informed me that they had been recommended by the government to visit a Dāī.

*Marvi*: The woman or her family makes these decisions. There is no role of the government. Moreover, no one was recommended by the government to visit me.

*Gulan*: The government is not in favor of home delivery. If women give birth at home, the number of coronavirus patients will decrease [and the hospital will make less money]; therefore, the government is interested in hospitals rather than home.

*Zainab*: I think the government encourages women to deliver their babies at a hospital. Yet, in my villages, I did not hear anything from women that they have been directed to visit a hospital. And there were some women who were conscious not to visit a hospital due to the risk of being infected.

#### Are you training apprentices to follow in your footsteps, or will your knowledge die with you?

Razul and Marvi are not training anyone; they say that no younger women want to be midwives, preferring more professional jobs. Sara is training her daughter-in-law, and Gulan is training her daughter “because I want our family to provide the services of Dāī till *Qayāmat* [the Day of Judgment]. When I die, hopefully, my daughter transfers this *Ddaihī* [Indigenous] knowledge to the coming generation.” Zainab wishes to train others, but states that “there are significant impacts of several maternity homes run by nurses in our area.” Yet she is sure that “no one can take away our right to lead a *Chathī* [again, a ritual arranged to name the baby on the sixth day], or to provide our postnatal services.

### Discussion and Analysis of Dāyūn’s Practices and Perceptions

In sum, the Dāyūn we interviewed are all older women with children, most of whom need the financial help that working as a Dāī provides them. They were all trained by older female relatives and some, like Gulan and Zainab, are determined to carry forward this multi-generational knowledge and skillset within their families. In contrast, others, like Marvi and Razul, understand that their knowledge will die with them because, as they noted, younger girls today are not interested in midwifery. Receiving no institutional support, these Dāyūn carry on as best they can. They charge little for their services and often receive even less; nevertheless, whatever they receive from families helps a great deal with their family income. And it also helps those families, as biomedical practitioners charge high prices for their services, including those who run the maternity homes—charges that can be avoided if the mother goes to a Dāī. All report that they are still highly respected in their communities, yet Razul noted that some now perceive Dāyūn as “illiterate people who know nothing.”

In general, their practices consist of prenatal massage, delivery attendance (which had been diminishing for some until COVID-19 sent more women to some of them), and postpartum care consisting of baby massage, washing bloody clothes considered highly polluting, helping with home chores, and leading the baby-naming ceremony—which, as Zainab points out, culturally cannot be taken away from them even if births are. Their delivery skills include the ability to turn the baby *in utero* into a better position for birth—something few, if any, hospital practitioners know how to do; they simply perform cesareans instead (Daviss and Bisits 2021). And it should be noted that most obstetricians today have lost the skills for attending vaginal breech birth (ibid.), while these Dāyūn are preserving them. In addition, “modern” homebirth midwives in high-resource countries, where homebirth rates hover between 1 and 2% and the usual number of births homebirth midwives attend per month is around 4 (personal correspondence with US midwives Vicki Penwell and Marimikel Potter, December 2020) would consider it extremely challenging to take on as many deliveries per month (often as many as 8) as these Dāyūn are accustomed to doing—although, during the coronavirus pandemic, many US homebirth midwives are doing exactly that (ibid).

Concerning COVID-19, like many in Sindh Province (Ali, 2020e; Ali and Ali, 2020), these Dāyūn perceive this disease as propaganda generated by the government to meet its vested interests, such as controlling the population and receiving additional foreign aid. Their belief or strong suspicion that COVID-19 is not real led them to take no extra precautions in addition to the ones they already used. These Dāyūn made it clear that whether the birth takes place in their homes or in the birthing women’s homes, they strive for cleanliness and wash their hands pre- and post-birth. Yet they are happy about this “non-real” COVID-19, as some of them have seen an uptick in clients (who do believe that is real) fleeing hospital contagion. Although they insist that they can handle birth complications, at the same time they seem fine with referring women to local maternity homes or clinics when the Dāī feels that the situation has gone beyond her ability to handle it.

It is beyond the scope of this article to investigate each and every practice these midwives describe. However, after a quick internet search, we can say that some of their remedies, which may seem ridiculous on the surface, do turn out to have scientifically demonstrated efficacy. For example, placing cotton pouches dipped in glycerin and mustard oil inside the uterus to prevent infection from a dead fetus may actually be effective, as mustard oil possesses powerful antimicrobial properties and may help block the growth of certain types of harmful bacteria. And cardamom is also an anti-bacterial and immune system booster, while castor oil, which has strong anti-inflammatory effects, has long been used by US homebirth midwives to help induce labor—a practice initiated as far back as ancient Egypt. A randomized controlled trial found that “Castor oil is effective for labor induction, in post-date multiparous women in outpatient settings” (Gilad et al., 2018:1).

Cow urine, which Sara mentioned that she uses for neonatal resuscitation, has long been used in Ayurveda, as the cow is considered sacred in India, but we could find no evidence of its efficacy. We thought that perhaps this practice was to make the baby gag and therefore breathe, but according to experienced midwife Vicki Penwell (personal communication January 26, 2021):

Anything more solid than a liquid that is put far enough back to elicit a gag reflex would be a potential hazard to block the airway; it is contraindicated to put anything in the mouth of an unconscious person, unless you are actually inserting an airway or intubating... I can see no benefit and lots of potential harm in this practice.

In contrast, patting the baby’s back and delivering mouth-to-mouth resuscitation are generally effective for babies who do not immediately breathe. Waiting to cut the cord for 1–2 min, as almost all of these Dāyūn do, is consistent with international guidelines on delayed cord clamping, which allows more oxygenated blood to flow from the placenta to the newborn. Yet placing an old, though “clean,” cloth or piece of rug into the uterus to stop post-partum hemorrhage (PPH) is a dangerous practice that can produce infection. Abdominal massage for stopping PPH is indeed helpful, yet having the mother drink cold water or walk to stop PPH are unproven and likely ineffective techniques—walking especially is likely to increase the bleeding—unless the bleeding is due to a retained placenta and walking helps it to come out. (We did not specifically ask what the Dāyūn do for retained placentas.) Yet all our Dāyūn interlocutors do try to bring hemorrhaging women to a biomedical facility if they themselves cannot stop the bleeding, although sometimes, as Sara described, the Hindu family does not allow it if the Pothī ritual says “no,” demonstrating the strong influence that religious beliefs can have on birth.

Certainly, baby massage is likely to help the infant sleep. As for the rest of their practices, such as placing something heavy on the mother’s abdomen to “bring the uterus to its regular place and reduce belly fat,” and invoking Allah or a certain goddess and performing certain rituals, these are culturally embedded and meaningful to both the Dāyūn and their clients. Thus, the care they provide is socioculturally, if not always medically, safe. For example, ritually invoking the help of a goddess in whom all present believe can help to replace fear with a sense of safety and control, as rituals are so good at doing (Davis-Floyd and Laughlin, 2016).

Yet clearly, the practices of these Dāyūn are a mixed bag regarding medical efficacy. We recommend further research on the efficacy of traditional midwives’ practices everywhere before they are gone, as around the world, they are being phased out of practice or dying without passing on their knowledge and skills to future generations. For as we have shown, some of their practices are indeed efficacious and could be useful to contemporary practitioners. For a bit of cross-cultural comparison, traditional midwives in Mexico have for centuries rubbed the mother’s own birth blood onto her belly to stop a post-partum hemorrhage (Davis-Floyd, 2018), and professional Japanese independent midwives use a turkey baster to inject that blood into her rectum for quicker absorption (ibid.)—both of which may seem as ridiculous at first glance as some of the practices of the Sindh Dāyūn—until one realizes that this blood contains high levels of oxytocin, which helps the uterus clamp down and stop the bleeding. Thus, it is clear that such practices should be investigated for possible efficacy, rather than simply being dismissed as vestiges of an outdated past.

## Mothers’ Perceptions of the Dāyūn

The excerpts and information we present herein from our 11 interviews with mothers (whose names are anonymized) are designed to provide sociocultural context for the practices and cultural positionings of the Dāyūn of Sindh; thus they are focused on these mothers’ beliefs about and usages or non-usages of Dāyūn. Some of these mothers emphatically preferred delivery with a Dāī. Two of them, both of whom lived in a small village with some formal education (which their husbands also had) and limited resources, were each planning to deliver their fifth child in their homes assisted by a family Dāī, as they had previously done. They stated, “Our Dāī Aman is caring and knowledgeable. She has not only assisted us but has also assisted our mothers and grandmothers in delivering all their children.” One of these women noted that her mother-in-law had birthed 10 babies with the assistance of a Dāī Aman. And one woman with a Master’s degree stated that she visits a hospital for antenatal care (ANC) but prefers to be assisted by a Dāī for labor and birth during COVID-19 because she fears hospital infection. Another woman, 30 year-old Sumbal, who received no formal education, had recently lost her baby during ANC. She shared her painful story, “I was interested in giving birth at a hospital but due to careless doctors, I lost my baby [prenatally]. It would have been better to stay at home. Now in the future, I will never go to a hospital for any prenatal care [or for birth, but will use my local Dāī Aman].”

In contrast, Sania, who has a grade 12 education and a husband who is a government employee, stated that she did not believe in the Dāyūn “because they are not fully trained.” Likewise, Sumaira, who is in her 30 s with a Master’s degree and has a private job and a self-employed husband, delivered her first two babies at home with a Dāī, but found that these births were “very painful,” so she chose a maternity home run by “an intelligent and caring” nurse, whom she called a *Dākdāryānnī* (a term used for a female doctor) for her next three deliveries. The last one was during the pandemic, and the nurse “took extra care. She was wearing a mask and cleaning her hands repeatedly,” but she did not require Sumaira to wear a mask “because it was difficult for me to breathe.” Yet after the delivery, Sumaira “called our family Dāī Aman to massage my baby and me as well as washing my clothes and arranging the ritual of *Chathī.* We paid her around US$30, including some food and the money people paid during *Chathī*. I still think to pay her more.” Yet Sumaira did not trust this Dāī to deliver a baby, saying:

Recently, one woman lost her son due to this Dāī’s inappropriate handling. When it was not possible anymore to assist the delivery, she brought the woman to the nearby maternity home where I deliver my babies. The nurse was shocked because it was not possible to save the baby, and there were risks involved that the mother might die, too. The nurse [did her best and] luckily, the mother survived.

Similarly, she mishandled another mother and brought her to the same nurse when the mother's situation was already too complicated. The nurse saved the mother and the baby. However, the mother died after almost three weeks. The underlying reasons in both cases were that she [the Dāī] uses some medicine that she puts in the *Bbachydānī* [this term is used interchangeably for vagina and uterus]….My mother and grandmothers delivered their babies assisted by the grand generations of these Dāī, but they were more intelligent and skillful [than those of today].

What Sumaira says about the greater skills of the “grand generations” of these Dāyūn may well be true, as these former generations had no medical backup at all and so had to rely entirely on their own authoritative knowledge and skills in handling birth complications. In contrast, today’s Dāyūn know that they can refer clients with complications to the nearby medical facilities and that their knowledge and skills are denigrated by medical authorities and some members of their own communities, and thus their knowledge no longer counts as authoritative in the eyes of many.

In these mothers’ words and in the words of some of the Dāyūn whom we quote, we can detect the gradual demise of the Dāī in Pakistan. As maternity homes and hospitals become more readily available, a growing number of pregnant women are choosing this much more modern mode of care, and indeed, as Dāī Razul stated, many are now indeed viewing the Dāyūn as illiterate and premodern vestiges of the past.

## Study Limitations

This study has a specific limitation: due to word length requirements, we were not able to include the full results of all of our 11 interviews with the mothers. Nevertheless, these case studies provide unique insights into two largely unresearched arenas: the practices of the Dāyūn of Sindh, and the roles of the Dāī during the COVID-19 pandemic—which, in our study, turned out to have only to do with the ways in which they already practiced. And we trust that the limitation of our small number of interlocutors is counterbalanced by the first three local authors’ lengthy and detailed ethnographic research in the Province, especially on health, illness, vaccination, and, most recently, maternity care—all of which have informed the background and context we provide in this article.

## Conclusion: Recommending Training and Full Integration for the Dāyūn

In this article, we have presented the voices and choices of both Dāyūn and childbearing women regarding childbirth practices, facility birth, and the now globally syndemic COVID-19, placing primary focus on the voices of the Dāyūn and more limited focus on mothers’ perspectives on their practices. After briefly situating this article within Pakistan’s socio-cultural, economic, and political landscape, we have shown that the government has made substantial efforts to shape people’s perceptions and practices related to perinatal care and to COVID-19 by foregrounding the authoritative knowledge and the authority of biomedicine as practiced in Pakistan. And we have illustrated Pakistan’s medical pluralism, its lack of governmental support, and how the Dāyūn incorporate aspects of integrative modalities like Ayurveda and Unami-Tib, along with their own remedies as passed down to them through generations.

Although Dāyūn have historically attended the vast majority of births in the country, today pregnant women are increasingly choosing medical facilities for antenatal care and delivery as part of the modernizing process that is sweeping over low- and middle-income countries and that leads to the perception of traditional midwives as premodern vestiges of the past (Davis-Floyd, 2018). Yet as we have shown, a significant percentage of rural women in Pakistan still choose Dāyūn for delivery, and the Dāyūn maintain their status as providers of the valuable pre- and postnatal services described above, which include abdominal massage; turning the baby *in utero* (called “external version”)—a skill many obstetricians do not have; the administration of certain medicinal remedies with varying degrees of efficacy; the washing of clothes considered too impure for others to touch; baby massage; and leading the *Chathī*—the baby-naming ceremony.

As we have shown, Dāyūn learn their knowledge and skills from other Dāyūn, usually older family members, but seem very open to receiving formal training, although no such government training has been offered to our interlocutors (an NGO did offer one training, which one of them benefited from, thereby demonstrating the need for more). They assist delivery either at their home or at the mother’s. Out of the five Dāyūn we interviewed, two are teaching these skills to the younger generation, while the others recognize that their knowledge will die with them. These Dāyūn claim that they can handle all pregnancy- and birth-related complications, including vaginal breech deliveries, but clearly, given their statements that they do refer women to medical facilities, sometimes they need biomedical help—in addition to the supernatural help they seek via ritual performance. Some of their practices seem scientifically questionable, while others have clear benefits, as described above.

Unfortunately, it is not possible to know their statistical outcomes, as the government does not keep these; thus we have no way of quantitively assessing the results of their practices. Should such records begin to be kept, they might or might not show efficacy. Our five Dāyūn interlocutors shared that around 31 women have died over the decades of their care. Perhaps some or all of these deaths could have been prevented had the Dāyūn been given formal training. Yet statistics show that thousands of pregnant women die during their hospital deliveries. As noted earlier, the overall MMR in Pakistan is high, at 170/100,000, and cannot be blamed entirely on the Dāyūn, as they account for only 24% of the births in that country.

Regarding baby deaths, these Dāyūn report more than 40 among them, while some did not remember, so let’s guess high, around 80 in total. If we guess that on average, each Dāī has attended around 1,500 births (though some attended less and some more), that would be 7,500 lifetime births for our five Dāyūn with a perinatal mortality rate of 80 perinatal deaths per 7,500 births, or 10/1,000. That rate is far lower than the overall Pakistani perinatal mortality rate, which is 57/1,000 (NIPS and ICF, 2019).

Like many others in Sindh Province, these Dāyūn consider COVID-19 to be government propaganda, perhaps an attempt by the Pakistani government to secure more foreign funding. Since they do not take COVID seriously, they have not adapted their practices to using preventive measures nor PPE, beyond their normal practices of cleanliness. Nevertheless and somewhat ironically, they have seen an increase in the numbers of women seeking their services both for ANC and for birth in order to avoid hospital contagion. For that reason, these Dāyūn have welcomed the advent of this “fake” disease.

Given that in normal times, around 24% of Pakistani women, especially those living in rural areas, still prefer the Dāyūn for their perinatal care, and that during a pandemic, more pregnant women than usual seek their care, we stress that these Dāyūn should be given additional, formal training to better prepare them for successful birth attendance at all times. For example, although it is not possible to project an MMR from 31 maternal deaths out of 7,500 births, as the numbers are too low (MMRs are determined by X amount of deaths per 100,000), nevertheless 31 is a very high number for 7,500 births (Yet it is important to remember that many of the women these Dāyūn attend are malnourished and thus are often stunted and have weakened immune systems). It remains clear that these Dāyūn truly need advanced training to prevent maternal deaths—for example, in the administration of misoprostol/Cytotec to stop PPH after the baby is born and the placenta is out, as has been successfully done in Afghanistan by Canadian midwife Betty-Anne Daviss and her team (see Daviss, 2021) and in Ethiopia *via* the Home-Based Lifesaving Skills program (described in Buffington et al., 2021).

We must consider the numerous and critical roles of these Dāyūn, the ongoing preferences of many women for their care, and the acceptance of their knowledge as authoritative by many in their communities. We foreground these considerations within the contexts of the challenges posed by COVID-19 and the structural disparities of the country, which result in better biomedical care and better-equipped and staffed biomedical facilities for the urban yet not for the rural poor. (As we have shown, some of the biomedical clinics available in Sindh Province are staffed by only one person.) Thus we strongly suggest that the government, in addition to supplying these Dāyūn with the requisite skills and tools to better deal with birth complications, should also formally link them to a particular medical facility for client transfer when needed. And we suggest that this facility should welcome such transfers of care from Dāyūn and should encourage them to stay throughout labor and birth to provide culturally safe continuity of care (see Barclay, 2009 for a description of such a successful program in Samoa). This would provide what Davis-Floyd (2003b) has called “seamless articulation” during transport, as contrasted with the “fractured articulations” that occur when the transporting midwives are disregarded or actively shamed by biomedical personnel for not using a biomedical facility in the first place.

Instead of suffering from syndemic structural disparities in a health care system aligned against them and that fails to adequately serve the rural and Indigenous poor, thereby perpetuating the legacies of colonization (Foucault, 1973; Banerji, 1974; Banerji, 1981; Zaidi, 1988; Farmer, 1996; Singer and Baer, 2018; Ali, 2020a; Ali, 2020b; Ali, 2020c; Ali and Ali, 2020; Ali and Davis-Floyd, 2020; Ali et al., 2020), Dāyūn should become officially recognized and integrated frontline caregivers in their areas. This will help to lessen the overwhelming impacts on the insufficient healthcare system of the country during this present pandemic and future emergencies to come; will assist the economically poor to receive appropriate and affordable treatment; and will help these Dāyūn themselves to make a viable living. We strongly propose institutionalizing these practitioners at the grassroots level to improve their skills (thereby potentially saving lives) and to mitigate both the unnecessary over-medicalization and commodification of pregnancy and birth and the rising hegemony of the biomedical healthcare system and its system of authoritative knowledge. Integrating the Dāyūn, with their knowledge of “alternative” healthcare systems, would also help to facilitate the growth of medical pluralism in Pakistan and to reduce its population’s increasing dependency on biomedicine. Given that Ayurvedic medicine and Unami-Tib are efficacious enough to have gained popularity in many countries of the Global North (though they—and especially Ayurveda—are regarded by biomedical doctors and researchers as ineffective and even dangerous), it seems only reasonable that they should also be fostered in their countries of origin in the Global South, and that the home remedies of the Dāyūn that are efficacious should also be researched, recorded, and transmitted.

As previously noted, biomedicine is only one among many healing modalities, and it only makes sense that the authoritative knowledge systems of other, integrative modalities should be preserved, as the Pakistani Dāyūn are doing. Especially given that these “alternative” and “complementary” knowledge systems are far cheaper and much lower-tech and less carbon-intensive than Western-style biomedicine, we argue that they should be further integrated and augmented. In this Anthropocene Era, the onrushing Climate Crisis is poised to cause multiple disasters and future pandemics that may well overwhelm biomedical facilities and prove the necessity of flexible, community-based, “low-tech, skilled touch” (Davis-Floyd et al., 2021) models of care such as those provided by many community midwives around the world (ibid). The Dāyūn of Pakistan, if given the additional training they need and fully integrated into the healthcare system, can become just such providers, who will be greatly needed in the uncertain future to come.

## Data Availability

The datasets presented in this article are not readily available because the data are confidential. Requests to access the datasets should be directed to inayat_qau@yahoo.com.

## Footnotes

1 These questions are from an etic perspective, whereas to explore roles, perceptions and practices during ordinary and extraordinary Covidian times, we used a Sindhi version—the emic version—of the interview guide, which asked these questions in locally comprehensible terms.

2 Concerning the details of Chathī, please see Ali, 2020b.

3 These Dāyon as well as mothers use a term called *Bbār* that in English translates as “baby” not “fetus.”

4 Moreover, the word “operation” is also used for any small or large surgical biomedical intervention. Laypeople also use this term for women who get a tubal ligation to prevent them from conceiving in the interests of family planning.

5 They don’t call it “COVID-19” or a “pandemic.” Instead, they use the terms “corona,” “coronavirus,” or *Wabā* (an infectious disease). Concerning the language of the pandemic in Pakistan, please see Ali and Davis-Floyd, 2020.

6 In studying such rumors, Inayat Ali (2021b) has found that many people in Pakistan believe that “whoever goes to a hospital during coronavirus never returns alive.” Such rumors spread quickly in the country (see also Ali, 2020c; Ali, 2020e; Ali, 2021a).

7 Our interlocutors were unaware of the acronym “PPE.” Instead, they called it Hifāzatī Sāmān, which literally translates as “preventive stuff.”

8 These are government funded programs to support the economically poor.
